# Associations of sleep duration with physical fitness performance and self-perception of health: a cross-sectional study of Taiwanese adults aged 23–45

**DOI:** 10.1186/s12889-021-10636-9

**Published:** 2021-03-25

**Authors:** Ming Gu, Chia-Chen Liu, Chi-Chieh Hsu, Chi-Jie Lu, Tian-Shyug Lee, Mingchih Chen, Chien-Chang Ho

**Affiliations:** 1 Graduate Institute of Business Administration, Fu Jen Catholic University, New Taipei City, 24205 Taiwan; 2 Department of Physical Education, National Taichung University of Education, No.140, Minsheng Rd., West Dist., Taichung City, 40306 Taiwan (R.O.C.); 3 Department of Physical Education, National Taiwan Normal University, Taipei City, 10610 Taiwan; 4 Department of Aquatic Sports, University of Taipei, Taipei City, 11153 Taiwan; 5 Artificial Intelligence Development Center, Fu Jen Catholic University, New Taipei City, 24205 Taiwan; 6 Department of Information Management, Fu Jen Catholic University, New Taipei City, 242304 Taiwan; 7 Department of Physical Education, Fu Jen Catholic University, New Taipei City, 24205 Taiwan; 8 Research and Development Center for Physical Education, Health, and Information Technology, Fu Jen Catholic University, New Taipei City, 24205 Taiwan; 9 Office of Physical Education, Fu Jen Catholic University, New Taipei City, 24205 Taiwan

**Keywords:** Physical fitness, Functional fitness, Sleep duration, Obesity, Self-perception of health, Adults, Taiwan

## Abstract

**Background:**

The relationship between sleep duration and physical fitness is one aspect of sleep health. Potential factors associated with sleep duration interfere with physical fitness performance, but the impact trends on physical fitness indicators remain unclear.

**Methods:**

This study examined associations between sleep duration and physical fitness among young to middle-aged adults in Taiwan. A total of 42,781 Taiwanese adults aged 23–45 participated in the National Physical Fitness Examination Survey 2013 (NPFES-2013) in Taiwan between October 2013 and March 2014. A standardized structural questionnaire was used to record participants’ sleep duration, which was stratified as short (< 6 h/day (h/d)), moderate (6–7 h/d; 7–8 h/d; 8-9 h), and long (≥ 9 h/d) sleep duration groups. Physical fitness was assessed based on four components: body composition (body mass index [BMI], waist-to-height ratio [WHtR], and waist-to-hip ratio [WHR]), muscle strength and endurance (1-min bent-leg sit-up test [BS]), flexibility (sit-and-reach test [SR]), and cardiorespiratory endurance index (3-min step test [CEI]).

**Results:**

By using analysis of covariance (ANCOVA), after sex grouping and age adjustment, we observed that sleep duration was significantly associated with obesity, functional fitness, and self-perception of health. The sleep duration for low obesity-related values (BMI, WHtR, and WHR) for men was 7–9 h/d, and that for women was 7–8 h/d. Sleeping more than 8 h/d showed poor functional fitness performances (BS and SR). For both sexes, sleep duration of 8–9 h/d was the optimal sleep duration for self-perceptions of health.

**Conclusions:**

Our research found that there were wide and different associations of sleep duration with physical fitness and self-perception of health among Taiwanese adults aged 23–45, and there were differences in these associated manifestations between men and women. This study could be of great importance in regional public health management in Taiwan, and provide inspirations for clinical research on physical fitness.

## Background

In recent years, many studies have suggested associations between sleep duration and health status. Research on sleep and health has focused on many aspects, such as chronic disease, obesity, self-perception of health, etc. However, there were different opinions on the appropriate sleep duration. Several studies showed that, for adults aged 18–60, sleeping less than 7 h/day (h/d) was associated with increased risk for obesity, diabetes, hypertension, coronary heart disease, stroke, mental distress, and all-cause mortality [1–3]. Alvarez and Ayas [4] found that individuals who reported increased (> 8 h/d) and decreased (< 7 h/d) sleep durations had moderately increased risks of all-cause mortality, cardiovascular disease, and symptomatic diabetes. Gottlieb et al. [5, 6] revealed that sleep durations of ≤6 h/d and ≥ 9 h/d were associated with an increased prevalence of hypertension, diabetes and impaired glucose tolerance. Garaulet et al. [7] found that the optimal sleep duration was 8 h/d, and people with shorter sleep duration showed higher obesity risk, particularly in women. Cappuccio et al. [8] showed that the obesity risk consistently increased in adults who slept less than 5 h/d. Hsu et al. [9] found that 7 h/d of sleep duration was the recommended duration for good physical fitness performance. Steptoe et al. [10] found that self-rated health was poorer in respondents who slept < 7 h/d or > 8 h/d than in those who slept 7–8 h/d. Magee et al. [11] observed that short (< 6 h/d) and long (≥ 9 h/d) sleep durations were significantly associated with poor self-rated health and low quality of life. However, Watson et al. [12] explained that little empirical evidence existed to indicate that long sleep duration (≥ 9 h/d) causes adverse conditions among healthy adults exists. These results emphasized the importance of adequate sleep for physical and mental health.

Physical fitness, which can be more objectively measured than physical activity, is a powerful indicator of health status. Physical fitness included body composition and functional fitness, among which, functional fitness of coordination, endurance, power fitness, and cardiovascular systems were prioritized [13–17]. Crucial physical fitness indicators for adults include body mass index (BMI), waist-to-height ratio (WHtR), waist-to-hip ratio (WHR), 1-min bent-leg sit-up test (BS), sit-and-reach test (SR), and 3-min step test which reflects the cardiorespiratory endurance index (CEI). BMI, WHtR, and WHR indicate the probability of being overall obesity or central obesity; BS indicates lumbar and muscle endurance; SR indicates hamstring flexibility and tension of the movable range of the lower back joint or the toughness of the ligament; and CEI indicates cardiorespiratory endurance.

However, the link between physical fitness and sleep duration is still poorly understood, and there were few studies related to Taiwanese adults. In the current study, we discussed the distribution of short, moderate, and long sleep duration among young adults in Taiwan, and explored how long sleep duration was the optimal sleep duration for physical fitness and self-perception of health.

## Methods

### Data source

We reviewed the data from 42,781 Taiwanese adults aged between 23 and 45 years who participated in the National Physical Fitness Examination Survey 2013 (NPFES-2013) in Taiwan between October 2013 and March 2014. The National Physical Fitness Examination Survey (NPFES) was implemented by the Sports Administration, Ministry of Education (MOE-SA) in Taiwan, and the purpose was to investigate the annual physical fitness status of Taiwan residents. Participants in NPFES-2013 were recruited using convenience sampling at 46 test stations in 22 cities and counties in Taiwan. The research protocol comprised three phases: participating in a standardized interview, pretest health screening, and physical fitness tests. These data were recorded using a standardized structural questionnaire, which included items related to demographic characteristics, sleep behaviors, self-perceptions of health, anthropometric characteristics and physical fitness measurements. All tests and records were conducted by well-trained examiners, and informed consent was obtained from each participant after a full explanation of the survey. The data obtained comprised de-identified secondary data, which were released to the public for research purposes [18]. This study was approved by the Institutional Review Board of Fu Jen Catholic University (FJU-IRB C108006).

### Anthropometric measurements

Anthropometric indicators included body weight, height, waist circumference (WC), and hip circumference (HC). Body weight and height were recorded to the nearest 0.1 kg and 0.1 cm with an electronic height-weight scale; WC was measured to the nearest 0.1 cm with a flexible steel tape measure placed midway between the lowest rib and iliac crest; and HC was measured to the nearest 0.1 cm at the widest part of the hip region. The above measurements required the participants to take off their shoes and thick clothes before measurements, and to be measured at the end of the exhalation in the standing position.

### Physical fitness measurements

The following four physical fitness measurements were taken according to the governmental guideline of the MOE-SA in Taiwan [18]: body composition (body mass index [BMI], waist-to-height ratio [WHtR], and waist-to-hip ratio [WHR]), muscle strength and endurance (1-min bent-leg sit-up test [BS]), flexibility (sit-and-reach test [SR]) and cardiorespiratory endurance (3-min step test [CEI]).

#### Body composition

Body composition was assessed by BMI, WHtR, and WHR, where: BMI = body weight (kg)/height squared (m^2^), WHtR = WC (cm)/height (cm), and WHR = WC (cm)/HC (cm). The procedures and instruments for the anthropometric measures (i.e., body weight, height, WC and HC) have been previously described.

#### Muscle strength and endurance

Abdominal muscular strength and endurance were measured using a timed bent-leg sit-up test (BS). Participants laid down on a mat with knees bent at right angles and hands behind the head; the ankles were firmly held by a partner. The BS test result was measured as the repetitions within 1 min.

#### Flexibility

Flexibility was measured using a sit-and-reach test (SR), which was considered a useful field test to evaluate hamstring and low-back flexibility. Participants took off their shoes before the test and sat on an SR box with a measuring scale. The examiner instructed participants to bend body slowly and recorded the maximum distance reached by their fingertips in cm.

#### Cardiorespiratory endurance

Cardiorespiratory endurance index (CEI) was used as an evaluation indicator of cardiorespiratory fitness performance. In this test, it was measured using an adapted 3-min step test from the Harvard Step Test. Participants were assisted by the examiner and a metronome cadence for repeatedly stepping on and off a 35-cm step for 3 min. After stepping, the participants were immediately seated, and CEI was calculated using the post exercise recovery heartbeat counts in intervals of 1 to 1.5 min, 2 to 2.5 min, and 3 to 3.5 min. CEI was calculated by the formula: CEI = duration of step test (s) × 100/sum of heartbeat counts during the recovery period/2.

### Sleep duration assessment

A standardized structural questionnaire was used to record participants’ self-reported daily average sleep duration. We selected nodes with < 6 h/day (h/d) and ≥ 9 h/d to divide the short and long sleep duration categories. For the sleep duration of middle interval, we took into account the definition differences in the literature [1–12], and divided it into the moderate sleep duration interval I (Moderate-I) when the sleep duration was greater than or equal to 6 h/d and less than 7 h/d; divided it into the moderate sleep duration interval II (Moderate-II) when it was greater than or equal to 7 h/d and less than 8 h/d; divided it into the moderate sleep duration interval III (Moderate-III) when it was greater than or equal to 8 h/d and less than 9 h/d.

### Self-perception of health

The standardized structural questionnaire was also used to record participants’ self-perceptions of health status. The assessments included three questions, asking about self-rated health, subjective happiness, and life satisfaction. According to the feeling of self-health from bad to good, each question was filled in 1 point for feeling very bad, 2 points for feeling bad, 3 points for feeling normal, 4 points for feeling good, and 5 points for feeling very good. The self-reported health assessments of NPFES-2013 used the same 5 classification answers as in references [19–22].

### Statistical analyses

All research data were processed using R software of version 3.6.3 (R core team, Vienna, Austria). T-test analysis was performed to analyze sex interference. For physical fitness performances and self-perceptions of health, age-adjusted analysis of covariance (ANCOVA) was used for determining the differences between short, moderate, and long sleep duration groups. Due to the sample imbalance for the groups, the Scheffe test was used for post-hoc tests [23]. Values were presented as means and standard deviations, or frequency percentages. Statistical results were significant at *p* <  0.05.

## Results

After processing missing values and outliers for the self-reported variables (sleep duration, self-rated health, subjective happiness, and life satisfaction) of the applied data, 40,944 valid sample individuals were obtained. Table 1 showed the results of T-test analysis of the study subjects grouped by sex. All variables showed significant differences between sex groups, in which the performances of 1-min bent-leg sit-up (BS) and cardiorespiratory endurance index (CEI) of males were better than those of females, while the performance of sit-and-reach (SR) of females was better than that of males. Regarding the self-perceptions of health, men’s self-rated health feelings were better than women’s, while women’s subjective happiness and life satisfaction were better than men’s.

**Table 1:** T-test analysis grouped by sex

Variables	Males	Females	*p*
No. of subjects	20,844 (51%)	20,100 (49%)	
age	34.15 ± 6.38	34.53 ± 6.36	< 0.001
Height (cm)	172.04 ± 6.05	159.54 ± 5.66	< 0.001
Body weight (kg)	73.91 ± 11.65	57.38 ± 9.75	< 0.001
BMI (kg/m2)	24.95 ± 3.64	22.54 ± 3.64	< 0.001
WC (cm)	84.82 ± 9.45	74.52 ± 9.15	< 0.001
HC (cm)	97.90 ± 6.99	93.96 ± 7.43	< 0.001
WHtR	0.493 ± 0.055	0.468 ± 0.058	< 0.001
WHR	0.865 ± 0.058	0.793 ± 0.065	< 0.001
BS (reps/min)	31.33 ± 9.23	21.10 ± 9.58	< 0.001
SR (cm)	22.54 ± 10.37	27.39 ± 10.80	< 0.001
CEI	56.50 ± 9.74	55.04 ± 9.84	< 0.001
self-rated health	3.62 ± 0.77	3.58 ± 0.76	< 0.001
subjective happiness	3.73 ± 0.78	3.79 ± 0.77	< 0.001
life satisfaction	3.68 ± 0.78	3.75 ± 0.77	< 0.001

Notes: *BMI* body mass index; *BS* bent-leg sit-up test; *CEI* cardiorespiratory endurance index; *HC* hip circumference; *SR* sit-and-reach test; *WC* waist circumference; *WHR* waist-to-hip ratio; *WHtR* waist-to-height ratio

Values were expressed as means ± standard deviations

Table 2 presented the results of age-adjusted ANCOVA by sex. For males, the post-hoc test results showed that, compared with those who slept 7–9 h/day (h/d), the group with less than 7 h/d of sleep had higher body mass index (BMI), waist circumference (WC), waist-to-height ratio (WHtR) and waist-to-hip ratio (WHR) values, which meant that less than 7 h/d of sleep might be associated with higher performances of obesity indicators. Regarding the functional fitness performances (BS, SR, and CEI), the group with more than 8 h/d of sleep showed poorer performances than that with less than 8 h/d of sleep. For self-perceptions of health, the moderate sleep duration of 8–9 h/d was the optimal sleep duration for presenting the best self-rated health, subjective happiness, and life satisfaction.

**Table 2:** Age-adjusted ANCOVA grouped by sex

Variables	Daily Sleep Duration (Hours)					*p*	Scheffe Test
	Short (< 6 h)	Moderate-I (≥ 6 h & < 7 h)	Moderate-II (≥ 7 h & < 8 h)	Moderate-III (≥ 8 h & < 9 h)	Long (≥ 9 h)		
Males							
No. of subjects	1563 (8%)	6635 (32%)	7984 (38%)	4225 (20%)	437 (2%)		
Height (cm)	171.7 ±5.94	171.94 ±5.98	172.10 ±6.11	172.23 ±6.05	171.68 ± 6.08	0.001	II, III > S
Body weight (kg)	74.76 ±12.48	74.49 ±11.48	73.46 ±11.41	73.48 ±11.86	74.37 ±12.94	< 0.001	S, I > II, III
BMI (kg/m2)	25.31 ±3.78	25.17 ±3.57	24.80 ±3.61	24.75 ±3.66	25.22 ±4.11	< 0.001	S, I > II, III
WC (cm)	85.63 ±10.19	85.18 ±9.37	84.45 ±9.24	84.58 ±9.54	85.70 ±10.07	< 0.001	S, I > II, III
HC (cm)	98.37 ±7.57	98.16 ±6.99	97.74 ±6.79	97.61 ±7.10	97.96 ±7.42	< 0.001	S, I > II, III
WHtR	0.499 ±0.059	0.496 ±0.055	0.491 ±0.054	0.491 ±0.056	0.500 ±0.060	< 0.001	S, I > II, III
WHR	0.869 ±0.058	0.867 ±0.059	0.863 ±0.057	0.865 ±0.059	0.873 ±0.058	< 0.001	S, I > II, III
BS (reps/min)	31.68 ±8.98	31.72 ±9.36	31.43 ±9.01	30.64 ±9.46	28.88 ±9.58	< 0.001	S, I, II > III, L
SR (cm)	22.73 ±10.32	22.90 ±10.36	22.47 ±10.26	22.14 ±10.54	21.47 ±10.95	< 0.001	I > III
CEI	56.72 ±9.99	56.77 ±9.78	56.39 ±9.66	56.34 ±9.74	55.14 ±9.74	0.003	S, I > L
self-rated health	3.42 ±0.90	3.57 ±0.78	3.64 ±0.73	3.73 ±0.74	3.59 ±0.85	< 0.001	III > II, L, I > S
subjective happiness	3.58 ±0.91	3.68 ±0.78	3.75 ±0.75	3.83 ±0.77	3.76 ±0.83	< 0.001	III > II > I > S
life satisfaction	3.52 ±0.88	3.63 ±0.78	3.71 ±0.75	3.77 ±0.78	3.68 ±0.91	< 0.001	III > II > I > S
Females							
No. of subjects	1531 (8%)	5909 (29%)	7200 (36%)	4776 (24%)	684 (3%)		
Height (cm)	159.38 ±5.51	159.47 ±5.62	159.69 ±5.64	159.50 ±5.74	159.14 ±5.96	0.03	II > I
Body weight (kg)	58.48 ±10.92	57.35 ±9.60	57.24 ±9.40	57.27 ±9.82	57.47 ±11.09	< 0.001	S > I, II, III, L
BMI (kg/m2)	23.02 ±4.10	22.55 ±3.60	22.44 ±3.46	22.52 ±3.74	22.66 ±3.99	< 0.001	S > I, II, III, L
WC (cm)	75.17 ±9.86	74.49 ±9.08	74.19 ±8.78	74.73 ±9.28	75.31 ±10.57	< 0.001	S, I, III, L > II
HC (cm)	94.45 ±8.27	93.81 ±7.48	94.01 ±7.07	93.91 ±7.51	93.98 ±8.09	0.05	–
WHtR	0.472 ±0.063	0.468 ±0.058	0.465 ±0.056	0.469 ±0.059	0.474 ±0.067	< 0.001	S, I, III, L > II
WHR	0.795 ±0.067	0.794 ±0.065	0.789 ±0.064	0.795 ±0.067	0.800 ±0.073	< 0.001	S, I, III, L > II
BS (reps/min)	21.13 ±10.10	21.19 ±9.65	21.66 ±9.52	20.39 ±9.30	19.23 ±9.78	< 0.001	II > S, I, III, L
SR (cm)	27.59 ±11.06	27.73 ±10.81	27.67 ±10.66	26.67 ±10.82	26.07 ±11.15	< 0.001	I, II > III, L
CEI	55.47 ±10.46	55.03 ±9.75	54.95 ±9.58	55.14 ±9.97	54.45 ±10.98	0.17	–
self-rated health	3.41 ±0.84	3.53 ±0.77	3.61 ±0.73	3.68 ±0.74	3.52 ±0.83	< 0.001	III > II > L,I > S
subjective happiness	3.65 ±0.85	3.73 ±0.76	3.80 ±0.75	3.89 ±0.75	3.80 ±0.82	< 0.001	III > II, L, I > S
life satisfaction	3.60 ±0.85	3.71 ±0.77	3.77 ±0.74	3.85 ±0.76	3.72 ±0.83	< 0.001	III > II, L, I > S

Notes: *BMI* body mass index; *BS* bent-leg sit-up test; *CEI* cardiorespiratory endurance index; *HC* hip circumference; *L* long sleep duration; *M* moderate sleep duration; *S* short sleep duration; *SR* sit-and-reach test; *WC* waist circumference; *WHR* waist-to-hip ratio; *WHtR* waist-to-height ratio; *I* moderate sleep duration interval I (Moderate-I); *II* moderate sleep duration interval II (Moderate-II); *III* moderate sleep duration interval III (Moderate-III)

Values were expressed as means ± standard deviations

For females, Table 2 showed that the group with less than 6 h/d of sleep had a higher BMI, while the sleep duration of 7–8 h/d was shown as the optimal sleep duration for central obesity indicators (WC, WHtR, WHR) and BS. Similar to men, women who slept for less than 8 h/d performed better in terms of SR. 8–9 h/d of sleep was also the optimal sleep duration for women to show the best self-rated health, subjective happiness and life satisfaction feelings. However, women’s CEI performance was not related to sleep duration.

## Discussion

Numerous studies have demonstrated the health benefits of sufficient sleep, but few studies have examined the relationship between sleep duration and physical fitness indicators. The purpose of this research was to determine the associations of sleep duration with physical fitness performances and self-perceptions of health in Taiwanese adults aged 23–45. This young and middle-aged labor force group in Taiwan generally had the characteristics of regular sleep and mild sleep deprivation. We selected a more detailed sleep duration grouping based on literature comparison, and used ANCOVA method to perform an age-adjusted analysis under sex division. Since the body compositions and functional fitness performances between male and female groups showed universal differences, it was necessary to divide the applied data by sex grouping. The results revealed that sleep duration was significantly associated with obesity, functional fitness, and self-perception of health.

The results showed that men who sleep less than 7 h/day (h/d) might face higher values of overall obesity and central obesity indicators, while women who sleep less than 6 h/d might face a higher value of overall obesity indicator. 7–8 h/d of sleep was the optimal sleep duration for women to stay away from central obesity. From the results, we cannot completely determine that there was no association of more than 9 h/d of sleep with men’s overall obesity and central obesity, because the self-reported sleep durations of more than 9 h/d were very few in the study sample. As mentioned, there was a general lack of sleep in Taiwanese adults aged 23–45, such sample imbalance might affect the statistical results of the long sleep duration group. Previous research mainly explored the association between short sleep duration and obesity, but there were different conclusions on the cut-off point of short sleep duration. Garaulet et al. [7] concluded that the cut-off point of short sleep duration for obesity risk was 8 h/d. Grandner et al. [3] indicated the short sleep cut-off point for obesity risk was 7 h/d. Bjorkelund et al. [24] indicated the cutoff was 6 h/d. Cappuccio et al. [8] and Stranges et al. [25] found the cutoff was 5 h/d, etc. Our findings supported that among Taiwanese adults aged 23–45, the cut-off point of obesity risk for men was 7 h/d of sleep, and the cutoff of overall obesity for women was 6 h/d of sleep. Women’s central obesity had a low-risk sleep duration interval of 7–8 h/d.

The present study found that men who slept for more than 8 h/d showed poorer functional fitness (BS, SR, and CEI) performances. Women who slept for less than 6 h/d or especially more than 7 h/d had poorer performances in BS, while similar to men, women who slept more than 8 h/d performed poorer in SR. The associations might indicate that prolonged sleep makes the muscles slack for a long time, which is not conducive to the development of the muscle strength and endurance and the flexibility. Or on the contrary, people with better functional fitness may be more energetic, more resistant to fatigue, and don’t need too much sleep [26]. Previous studies [27–30] have found that people with poor sleep quality were more likely to have lower performances of functional fitness and athletic ability. But few researchers have studied the relationship between sleep duration and functional fitness. Our results were unique and applicable to Taiwanese adults aged 23–45, and could provide inspirations for clinical research on functional fitness such as functional degeneration of articular capsule or ligament, and stiffness of muscle-tendon or connective tissue [31, 32].

In our results, for both sexes, sleep duration of 8–9 h/d was the optimal sleep duration for presenting the best self-rated health, subjective happiness, and life satisfaction. Shankar et al. [33] found that compared to 7 h/d of sleep, those with shorter or longer sleep duration were more likely to report poor self-rated health. Steptoe et al. [10] found that sleep duration of 7–8 h/d was the optimal for presenting the best self-rated health. Magee et al. [11] observed that short (< 6 h/d) and long (≥ 9 h/d) sleep durations were significantly associated with poor self-rated health and low quality of life. It can be speculated that our results were more representative of sleep wellbeing self-efficacy in Taiwanese adults aged 23–45.

The present study has several advantages. First, the sample size was large, and the data were representative of Taiwanese adults aged 23–45. Second, according to the review of relevant literature, this study was the first to investigate the association between sleep duration and functional fitness among young to middle-aged adults in Taiwan. Meanwhile, we divided the sleep duration interval into five categories in more detail for comparison. However, this study has some limitations. First, the sample lacked individuals who reported more than 9 h of sleep. Such sample imbalance might lead to poor robustness to the statistical results of the long sleep duration group. Second, a causal relationship cannot be demonstrated because of the cross-sectional study design. But longitudinal study design was relatively difficult to practice in this research direction. Sleep duration is an experimental condition or goal which is difficult to control, since it is usually related to one’s habits and schedule. This research is an apt opportunity to clarify how sleep might be a cause of and a consequence of adiposity and fitness-enhancing physical activity. More in-depth exploration should require the coordination of clinical research and detailed sleep data monitoring, and correlate that with phased adiposity or fitness conditions to establish a temporal order. Third, the survey data eliminated the functionally disabled and physically unfit population. Fourth, the data on sleeping and self-perception of health was self-reported data which could be biased.

Future research may include several aspects. First, we suggest that the subsequent National Physical Fitness Examination Survey (NPFES) should consider a more informative questionnaire covering issues about profession, self-reported sleep quality, alcohol use, diet, psychological stress, and depression which can be used as new independent variables as well as confounders. Secondly, a suitable longitudinal study design can be developed for clinical research on functional fitness and sleep monitoring. Furthermore, the research may link sleep, physical fitness, and chronic diseases to conduct a more systematic study. Previous studies have linked physical fitness to diabetes, hypertension, and heart disease [34–37]. Hence, future study may explore the effects of sleep status on chronic diseases through the mediating role of physical health.

## Conclusions

This study analyzed the associations of sleep duration with physical fitness performances and self-perceptions of health among Taiwanese adults aged 23–45. According to the results, sleep duration was significantly associated with obesity, functional fitness, and self-perception of health. The appropriate sleep duration for men with low obesity-related values was 7–9 h/day (h/d), and that for women was 7–8 h/d. Sleeping less than 8 h/d showed better functional fitness (BS, SR, and CEI) performances in males, and better SR performance in females, while 7–8 h/d was the optimal sleep duration for BS for women. For both sexes, sleep duration of 8–9 h/d was the optimal sleep duration for self-perceptions of health. We infer that sleep duration may be a potential factor regarding the effects on physical fitness and self-perception of health.

## Data Availability

The computerized data sets of the National Physical Fitness Examination Survey (NPFES) were comprised of de-identified data, which were released by the Sports and Health Information Application Research Center of the MOE-SA for public research purposes. The data set analyzed during the current study was publicly available at https://isports.sa.gov.tw/Apps/EssayList.aspx? SYS = TIS&MENU_CD = M04&ITEM_CD = T06&MENU_PRG_CD = 4&ITEM_PRG_CD = 5, based on the research project application and IRB.

## Abbreviations

ANCOVA: Analysis of covariance
BS: 1-munite bent-leg sit-up test
BMI: Body mass index
CEI: Cardiorespiratory endurance index
HC: Hip circumference
SR: Sit-and-reach test
WC: Waist circumference
WHR: Waist-to-hip ratio
WHtR: Waist-to-height ratio
